# Aggressive Juvenile Trabecular Ossifying Fibroma in a 4‐Year‐Old: Case Report Bringing Diagnostic Challenges and Surgical Management

**DOI:** 10.1155/crid/1239016

**Published:** 2026-09-15

**Authors:** Brett Spenrath, Amanda Gruza, Rani Kanthan, Tony L. Ng, Felipe F. Sperandio

**Affiliations:** ^1^ College of Dentistry, University of Saskatchewan, Saskatoon, Canada, usask.ca; ^2^ Department of Pathology and Laboratory Medicine, University of Saskatchewan, Saskatoon, Canada, usask.ca; ^3^ Faculty of Medicine, The University of British Columbia, Vancouver, Canada, ubc.ca

**Keywords:** case report, juvenile trabecular ossifying fibroma, molecular diagnostics, multidisciplinary management, preoperative embolization

## Abstract

Juvenile trabecular ossifying fibroma (JTOF) is an uncommon benign fibro‐osseous neoplasm of the craniofacial skeleton that predominantly affects children and may clinically and radiographically resemble other aggressive lesions. We describe the case of a 4‐year‐old boy with a rapidly enlarging, painless maxillary mass. Imaging revealed a well‐defined expansile lesion with internal radiopaque foci and ground‐glass attenuation, extending into the nasal cavity and orbit. Histopathology showed immature trabecular bone with osteoblastic rimming in a fibroblastic stroma, along with aneurysmal bone cyst‐like changes. Molecular testing using a next‐generation sequencing (NGS) panel was negative for GNAS (Guanine Nucleotide binding protein, Alpha Stimulating activity) mutations, aiding in the exclusion of fibrous dysplasia and supporting the diagnosis of JTOF. Notably, this case represents the use of preoperative embolization in JTOF management, which was successfully performed prior to total hemi maxillectomy. Postoperative CT scans demonstrated no evidence of recurrence through 16 months of follow‐up. A multidisciplinary approach was essential to the patient′s recovery, with pediatric dental collaboration enabling the fabrication of a custom denture that restored jaw function and improved quality of life. This report underscores the diagnostic challenges associated with JTOF and highlights the value of integrating molecular testing, individualized surgical planning, and coordinated multidisciplinary team care to achieve favorable outcomes in managing this uncommon pediatric lesion, which included involving a pediatric dentist to fabricate a custom denture to support functional recovery and improve the patient′s quality of life.

## 1. Introduction

Juvenile trabecular ossifying fibroma (JTOF) is a rare benign fibro‐osseous neoplasm that primarily involves the craniofacial bones of children and adolescents. It represents one of the two recognized juvenile variants of ossifying fibroma, together with the juvenile psammomatoid ossifying fibroma (JPOF). Although both lesions are characterized by locally aggressive behavior and a predilection for younger individuals, they differ in their typical age of presentation. JTOF most commonly develops between the ages of 8.5 and 12 years, whereas JPOF tends to occur later, with a peak incidence around the age of 20 [1]. JTOF normally arises in the maxilla or mandible, with a predilection for the maxilla, and can present as a rapidly expanding, painless mass that may lead to significant facial asymmetry and functional impairment [2]. Because of its clinical and radiographic similarities with other fibro‐osseous and osteogenic lesions [3], an accurate diagnosis is critical to guiding appropriate management and preventing unnecessary interventions.

The radiographic features of JTOF commonly include a well‐circumscribed, mixed radiolucent‐radiopaque lesion with variable degrees of peripheral cortication [4]. In many cases, tooth displacement, cortical thinning, and expansion are evident, and although root resorption and cortical perforation can occur, they are encountered less frequently [4]. The lesion′s aggressive growth pattern can sometimes mimic a malignant process, raising diagnostic challenges that necessitate thorough histopathological evaluation [3]. Microscopically, JTOF is distinguished by the presence of trabecular woven bone within a highly cellular fibroblastic stroma, often exhibiting osteoblastic rimming and, in some cases, areas of hemorrhagic or cystic degeneration [5].

We report the case of a 4‐year‐old boy who presented with an intrabony expansile lesion in the right maxilla. The lesion was detected due to progressive facial swelling, and imaging studies revealed a well‐defined radiolucent area with internal radiopaque foci, suggestive of a fibro‐osseous lesion. Given the patient′s age, rapid lesion growth, and radiographic findings, a provisional diagnosis of JTOF was considered, prompting further histopathological and molecular investigations to exclude other fibro‐osseous conditions and osteogenic neoplasms.

This case highlights the importance of considering JTOF in the differential diagnosis of pediatric maxillary lesions and underscores the vital role of a multidisciplinary approach in achieving an accurate diagnosis, also by presenting a tailored treatment towards achieving optimal rehabilitative outcomes with jaw function and aesthetics restoration. In the present case, collaboration with a pediatric dentist was instrumental to include the fabrication of a custom denture that supported functional recovery and improved the patient′s quality of life. Furthermore, we discuss the management strategies for JTOF, emphasizing the need for a balance between surgical intervention and preservation of facial aesthetics. Through this report, we aim to contribute to the growing body of literature on JTOF and provide insights into its clinical presentation, diagnostic workup, and long‐term management.

## 2. Case Report

A 4‐year‐old male was referred to the Oral and Maxillofacial Surgery clinic for evaluation of a mixed radiopaque/radiolucent mass in the right maxilla. Clinical examination revealed vertical dystopia and exophthalmos of the right globe, along with right cheek swelling. Extraocular movements and pupillary reflexes were intact, and there were no complaints of diplopia or visual disturbance. The medical history was unremarkable.

**Figure 1:**
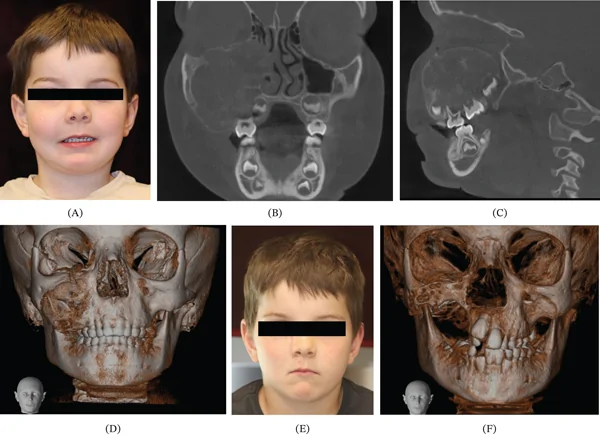
(A) Preoperative frontal repose photograph showing swelling of the right cheek. (B) Preoperative CT (coronal) imaging of facial bones with contrast showing a 4.7 × 3.9 × 3.5 cm expansile, well delineated lesion arising from the right maxilla with internal groundglass attenuation. (C) Preoperative CT imaging (sagittal) imaging. (D) Preoperative 3D reconstruction. (E) Postoperative frontal repose photograph showing normal facial expression without facial swelling and linear scar at the site of surgical intervention at 12 months. (F) Postoperative 3D reconstruction status post right total maxillectomy and post embolization of right internal maxillary artery showing no evidence of the previously seen right maxillary lesion through 16 months of follow‐up; the remaining paranasal sinuses are clear.

**Figure 2:**
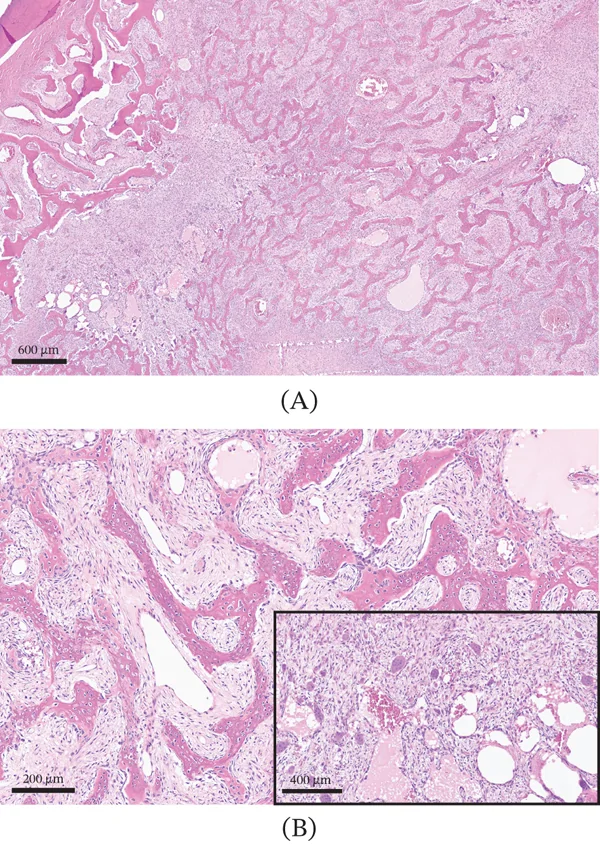
(A) Histopathological analysis (H&E—50X) showing islands of immature bone trabeculae with osteoblastic rimming along with some newly formed and elongated osteoid strands (“brush stroke” pattern) merging gradually with the surrounding stroma. The stroma consists of fibroblastic spindle cells with some dilated blood vessels. The stromal cells showed no significant nuclear atypia nor increase in mitotic count or necrosis. (B) (H&E—200X) Interconnected trabeculae of woven bone showing a moderate to hypercellular fibroblastic stroma, osteoblastic rimming and scattered clusters of osteoclastic giant cells. Inset. (H&E—100X) aneurysmal bone cyst‐type changes with conspicuous presence of giant cells.

Contrast‐enhanced CT of the facial bones demonstrated a 4.7 × 3.9 × 3.5 cm expansile, well‐circumscribed mass originating from the right maxilla. The lesion showed internal ground‐glass attenuation with extension into the right zygoma, orbital floor, and nasal cavity, displacing the globe superiorly (Figure 1). A transoral biopsy revealed immature bone trabeculae rimmed by osteoblasts with areas of osteoid formation (Figure 2). MDM2 (mouse double minute 2 homolog) immunostaining was interpreted as negative in the stromal tissue and did not warrant further testing, for example, FISH analysis. NGS panel was conducted via targeted sequencing on the Illumina MiSeq (see details below for full report) and was unremarkable. Based on the clinical, radiographic, histopathologic and molecular findings, a diagnosis of JTOF was established.

Surgical management involved a right maxillectomy including the orbital floor, lateral nasal wall, and alveolus. Considering the patient′s young age and remaining facial growth, a partial reconstruction was planned using split calvarial bone grafts to restore the orbital floor, infraorbital rim, and anterior maxillary sinus wall. Preoperative embolization of the right maxillary artery was performed, followed by tumor extirpation via a Weber–Ferguson incision with subciliary extension. The nasolacrimal system was preserved. Calvarial bone grafts were harvested and stabilized with resorbable plates and screws, whereas a temporalis myofascial flap provided soft‐tissue coverage. The patient tolerated the procedure well, was extubated intraoperatively, and recovered uneventfully in the pediatric intensive care unit (ICU).

Histopathologic examination of the resected specimen confirmed JTOF with pseudocystic degeneration and negative surgical margins. Postoperative cone beam computed tomography (CBCT) scans at 3, 6, and 9 months revealed no recurrence, with thickening of the anterior maxillary wall consistent with graft incorporation (Figure 1). A removable partial denture was provided for interim occlusal function and to prevent overeruption of the mandibular teeth. The patient demonstrated good orbital symmetry without eyelid malposition, although mild hypertrophic scarring was noted, planned for later revision. Definitive reconstruction with a vascularized osteocutaneous flap will be considered after completion of facial growth.

Full NGS panel results are as follows: mutation status: negative; mutation(s) detected: none; MSI status: MS‐stable; MSI score: 0.01. The reportable MSI statuses are as follows: MSI‐H (score > 0.6); equivocal (0.4 < score ≤ 0.6); and MS‐stable (score ≤ 0.4). Primers designed and validated to detect the following hotspots and exons are included in the assay: AKT1 (E17); ALK (T1151, L1152, C1156, F1174, L1196, L1198, 61202, D1203, S1206, G1269, R1275, Y1278); AR (L702, V716, S741, N742, 2784, H875, 5877, T878, M896); BRAF (Q201, G464, G466, F468, G469, Y472, D594, F595, G596, L597, V600, K601, K601‐M620, G606); CTNNB1 (D32, S33, 034, 135, H36, S37, T41, S45); DDR2 (L239, 1638, S768); DICER1 (D1705, D1709, G1809, D1810, E1813): EGFR (R106, A289, 5492, P596, G598, S709, L718, G719, L747, A750, K754, S768, T790, L792, G796, C797,L798, L833, L838, L858, L861, Exons 18‐21); ERBB2 (G309, S310, K753, L755, F767, D769, V773, G776, V777, Exon 20); ESR1 (K303, E380, S463, V534, P535, L536, Y537, D538); FGFR1 (N546, K656); FGFR2 (S252, P253, W290, A315, S372, Y375, C382, N549, K659, E731, E777); FGFR3 (R248, S249, G370, 5371, Y373, G380, A391, K650); FOXL2 (C134); GNA11 (Q209); GNAQ (Q209); GNAS (R201); HRAS (G12, G13, Q61); IDH1 (R132); IDH2 (R140, R172); KIT (S476, K550‐V555, X553, W557, V559. V560, L576, K642, V654, T670, D816, D820, N822, Y823, A829, Exons 9, 11, 13); KRAS (K5, A11, G12, G13, L19, 222, A59, G60, Q61, K117, A146); MAP2K1 (F53, 056, K57, K59, V60, D67, I103, I111, C121, N122, P124, P387); MAP2K2 (F57, Q60, K61, L119, H123, G132); MET (T1010, V1110, H1112, V1206, L1213, D1246. Y1248, Y1253, Exons 13. 14, 18); NRAS (G12, G13, A59, G60, Q61, K117, A146); NTRK1 (F589, G595, G667); NTRK3 (G623, G696); PDGFRA (R560‐E571, V561, P577, N659, L839‐Y849, D842, H845, D846); PIK3CA (R88, C90, R93, P104, G106, N107, R108, K111, R115, N345 I354‐P377, R357, G364, E365, C420, E453, P539, E542, E545, Q546, D549, 5978, M1043, N1044, A1046, H1047, G1049); POLE (P286, M295, 5297, F367, $36B, V411, L424, M444, A456. S459); PTCH1 (W844, G1093); PTEN (A126. C129. R130, R173 R233, K254‐K267); RET (G533, K603, C609, C611, C618, C620, C630, D631, C634, G691, E768, L790, Y791, V804, X806, A883, R886, S891, S904, M918, A919, Exons 10, 14, 15); ROS1 (S1986, L2026, G2032); STK11 (Q37, P281); TP53 (P72fs, L121ts, C135, P151, R158, A159, Y163, R175, L194, I195, S215, Y220, Y234, C238, S241, R249, D259, R273, P278, R280, Exons 2‐11).

### 2.1. Assay Limitations

Single nucleotide changes, insertions, and deletions at the targeted hotspots and exons will be reported. This assay does not assess gene expression. Single nucleotide variants, insertions and deletions have been validated to d5% variant allelic frequency (VAF%). Reporting variants below this threshold may be done at the discretion of the medical director. Rare genetic variation can interfere with this assay. The ability to detect a particular variant in a given specimen will depend upon the allele proportion of the variant in the extracted DNA combined with the lower limit of detection of the assay. This assay does not differentiate between germline and somatic mutations. This assay does not detect copy number variation (CNV) including amplification of HER2/neu nor does it detect gene fusions such as those leading to ALK gene rearrangements.

## 3. Discussion

JTOF is a rare fibro‐osseous neoplasm that predominantly occurs during childhood and adolescence, with an average age of onset around 11 years; in fact, fewer than 20% reported cases involve patients older than 15 [4, 6]. Therefore, presentation at the age of 4 years, as observed in the present case, is distinctly uncommon, although a maxillary JTOF has been described in a 2‐year‐old child [2]. Interestingly, discrepancies in diagnostic criteria may influence the reported age distribution, and it is likely that most classic JTOF cases arise before the 10 years of age. Current evidence indicates no clear sex predilection for this lesion [4, 6].

Clinically, JTOF usually leads to painless but rapidly enlarging swelling of the jaws, often accompanied by tooth displacement [7]. When the lesion occurs in the maxilla, it may lead to nasal obstruction and intermittent epistaxis [5]. Typical imaging findings of JTOF include well‐circumscribed lesions that appear radiolucent or exhibit a combination of radiolucent and radiopaque areas, frequently surrounded by a corticated margin [4]. In addition, the radiolucent pattern is more often unilocular than multilocular [4]. Despite these typical findings, the radiographic presentation is often nonspecific and may overlap with a variety of unilocular radiolucencies or mixed radiolucency/radiopacities [8], such as craniofacial fibrous dysplasia, odontogenic cysts and tumors, such as an odontogenic keratocyst, or a giant cell granuloma, which usually tends to present in a more lytic fashion.

Accordingly, fibrous dysplasia is consistently among the principal entities considered in the differential diagnosis of JTOF and can present in polyostotic or monostotic forms. JTOF cannot be confidently distinguished from fibrous dysplasia by imaging findings alone, and the benign fibro‐osseous histopathological appearance may also show overlapping features that make the diagnostic assessment challenging. This case brings interesting insights on how fibrous dysplasia was considered in the histopathological differential diagnosis; however, the abundance of trabecular osteoid production with osteoblastic rimming argued against it. In addition, GNAS mutation testing using an NGS panel was negative, further helping to exclude fibrous dysplasia. In addition, MDM2 immunohistochemical staining was interpreted as negative in the stromal tissue (a known pitfall of MDM2 immunostaining relates to strong positivity in macrophages/histiocytes [9]), and that was taken into consideration when analyzing our samples. Currently, there is no definitive diagnostic molecular marker for JTOF [3]. Rare examples of both juvenile ossifying fibroma and extragnathic psammomatoid FD have been reported to harbor amplifications involving MDM2 and RASAL1 (RAS protein activator like 1) [10]. Nevertheless, MDM2 overexpression and amplification remain exceptionally uncommon in OF and FD, and its identification should prompt careful consideration of low‐grade gnathic osteosarcoma in the differential diagnosis [11].

From a histopathological perspective, JTOF usually stands out from cemento‐ossifying (COF) and psammomatoid ossifying fibromas (PsOF) given its distinctive brown wavy strands that arise from garland‐like, curvilinear arrangements of edema, hemorrhage, osteoclasts, and pseudocystic degeneration [7]. In addition, PsOF typically shows small calcifications termed psammoma bodies, which are lacking in the present case [12]. Indeed, a trabecular woven bone pattern can be present in COF and other fibro‐osseous lesions; however, this characteristic alone is not sufficient for a definitive diagnosis. Although JTOF, COF, and PsOF share similarities, different anatomical and demographic characteristics are usually helpful to distinguish among them [12]. The histopathological presentation may also show overlap with low‐grade osteosarcoma [13]; however, the absence of atypical plump osteoblasts or tumor osteoid favored against that diagnosis. Likewise, hemorrhagic cystic degeneration may closely resemble an associated aneurysmal bone cyst (ABC) [4], further increasing the diagnostic complexity.

JTOF is a benign entity but does have a significant risk of local recurrence. One systematic review estimated an overall recurrence rate of approximately 21%, with recurrence reaching 53% after enucleation or curettage alone, compared with only 4% following marginal or segmental resection [4]. In addition, JTOFs can expand rapidly and lead to intracranial involvement; therefore, they should be treated surgically in an expedited manner aiming at complete resection [14]. Notably, this case highlights a successful management strategy for JTOF, demonstrating no recurrence over long‐term clinical and radiographic follow‐up. In fact, although a 16‐month follow‐up is valuable, long‐term surveillance should be advocated for JTOF given its known recurrence potential. Moreover, a multidisciplinary approach is crucial for achieving optimal rehabilitative outcomes in JTOF, particularly in restoring jaw function and aesthetics. In this case, collaboration with a pediatric dentist was instrumental, including the fabrication of a custom denture to support functional recovery and improve the patient′s quality of life. When an intraoral appliance is considered, mastication, speech adaptation, and functional rehabilitation need to be reassessed on a regular basis.

Preoperative embolization has previously been described as an adjunctive measure in the management of ossifying fibromas involving the paranasal sinuses, particularly after biopsy procedures complicated by substantial hemorrhage [15]. During the past two decades, selective embolization of feeding vessels has become increasingly incorporated into the treatment of highly vascular tumors of the head and neck, including paragangliomas and juvenile nasopharyngeal angiofibromas (JNAs) [15]. Effective management of intraoperative hemorrhage is particularly important in pediatric patients, since substantial blood loss can contribute to significant perioperative complications [16]. Preoperative embolization is a well‐established approach to decrease bleeding during surgery and has been successfully utilized in the context of pediatric JTOF [17]. Although endovascular procedures are generally safe, known complications can still arise as a consequence of reflux of embolic material or inadvertent migration through unrecognized collateral pathways between the internal and external carotid arteries. Notably, OFs, whether associated with ABC‐like changes or not, are frequently highly vascular and may have a close relation to the ophthalmic arterial network [15]. Given these anatomical considerations and the availability of modern hemostatic agents, one must carefully weigh the risks and benefits of preoperative embolization aiming at treatment individualization.

Despite the potentially aggressive local behavior and considerable size that JTOF may attain, malignant transformation has not been reported. This distinction is particularly important when differentiating it from fibrous dysplasia, for which malignant transformation, although exceedingly rare, has been documented [18]. In this context, molecular analysis for the presence of GNAS mutations is essential for accurate differentiation and may significantly influence the surgical approach and overall management strategy. In that sense, the use of an NGS panel in this case played a crucial role in confirming the diagnosis of JTOF and excluding fibrous dysplasia. The absence of this mutation in our case provided further diagnostic confidence, reinforcing JTOF as the definitive diagnosis and guiding appropriate management. This highlights the value of incorporating molecular testing into the diagnostic workflow for challenging fibro‐osseous lesions.

## Author Contributions

**Brett Spenrath:** data curation, formal analysis, resources, writing – original draft. **Amanda Gruza:** formal analysis, investigation, writing – original draft. **Rani Kanthan:** formal analysis, resources, writing – original draft. **Tony L. Ng:** formal analysis, resources, software, validation. **Felipe F. Sperandio:** conceptualization, data curation, formal analysis, investigation, project administration, supervision, writing – original draft, writing – review and editing.

## Funding

No funding was received for this manuscript.

## Disclosure

All authors have read and approved the final version of the manuscript. The corresponding author had full access to all of the data in this study and takes complete responsibility for the integrity of the data and the accuracy of the data analysis.

## Ethics Statement

Ethical approval is not required as it represents a single anonymized case report with informed consent. Written informed consent was obtained from the patient′s legal guardian. Institutional review board approval was not required in accordance with local regulations.

## Consent

Explicit written consent to publish the patient′s clinical details and photographs was given by the patient′s parents.

## Conflicts of Interest

The authors declare no conflicts of interest.

## Data Availability

The data that support the findings of this study are available on request from the corresponding author. The data are not publicly available due to privacy or ethical restrictions.
